# Diffuse Gastrointestinal Polyposis in Bannayan-Riley-Ruvalcaba Syndrome: A Rare Phenotype Among Phosphatase and Tensin Homolog Hamartoma Tumor Syndromes

**DOI:** 10.7759/cureus.18543

**Published:** 2021-10-06

**Authors:** Ivania Salinas, Genesis Perez Del Nogal, Alejandro Herrera, Pedro Rojas, Kejal Shah

**Affiliations:** 1 Internal Medicine, Texas Tech University Health Sciences Center, Odessa, USA

**Keywords:** general gastroenterology, cowden syndrome, colonoscopy, gastroenterology and endoscopy, hamartomatous polyp, polyposis, bannayan-riley-ruvalcaba syndrome, pten gene mutation

## Abstract

Bannayan-Riley-Ruvalcaba syndrome (BRRS) is a rare genetic disorder caused by germline mutations in the phosphatase and tensin homolog (PTEN) gene. Clinical manifestations arise early during childhood and include multiple lipomas, hamartomatous intestinal polyps, macrocephaly, developmental delay, and autism spectrum disorder among others. The case describes a 24-year-old female with a recent diagnosis of BRRS who presented for evaluation of burning epigastric pain for the previous six months. The esophagogastroduodenoscopy (EGD) and colonoscopy revealed an erosive gastric mucosa as well as numerous polyps throughout the gastrointestinal tract. Histopathologic examination confirmed gastric *Helicobacter pylori *infection and different histologic types of polyps.

## Introduction

Bannayan-Riley-Ruvalcaba syndrome (BRRS) is an autosomal dominant disorder caused by a germline mutation in a tumor suppressor gene called phosphatase and tensin homolog (PTEN). Clinical findings include macrocephaly, developmental delay, autism spectrum disorder, multiple lipomas, and hamartomatous intestinal polyps among others [1]. This syndrome (of unknown prevalence) belongs to a group of rare disorders collectively known as PTEN hamartoma tumor syndromes (PHTS) [2]. Recent data have shown that intestinal polyps are found in up to 95% of adults with PTEN mutations who have undergone colonoscopy. The range of polyp types includes ganglioneuromas, inﬂammatory polyps, and lymphoid polyps. The lifetime risk of developing colorectal cancer among patients with PTEN mutations is 9%. We describe the gastrointestinal findings observed in a patient with BRRS and discuss the current management and the recommended cancer surveillance guidelines.

## Case presentation

**Figure 1:**
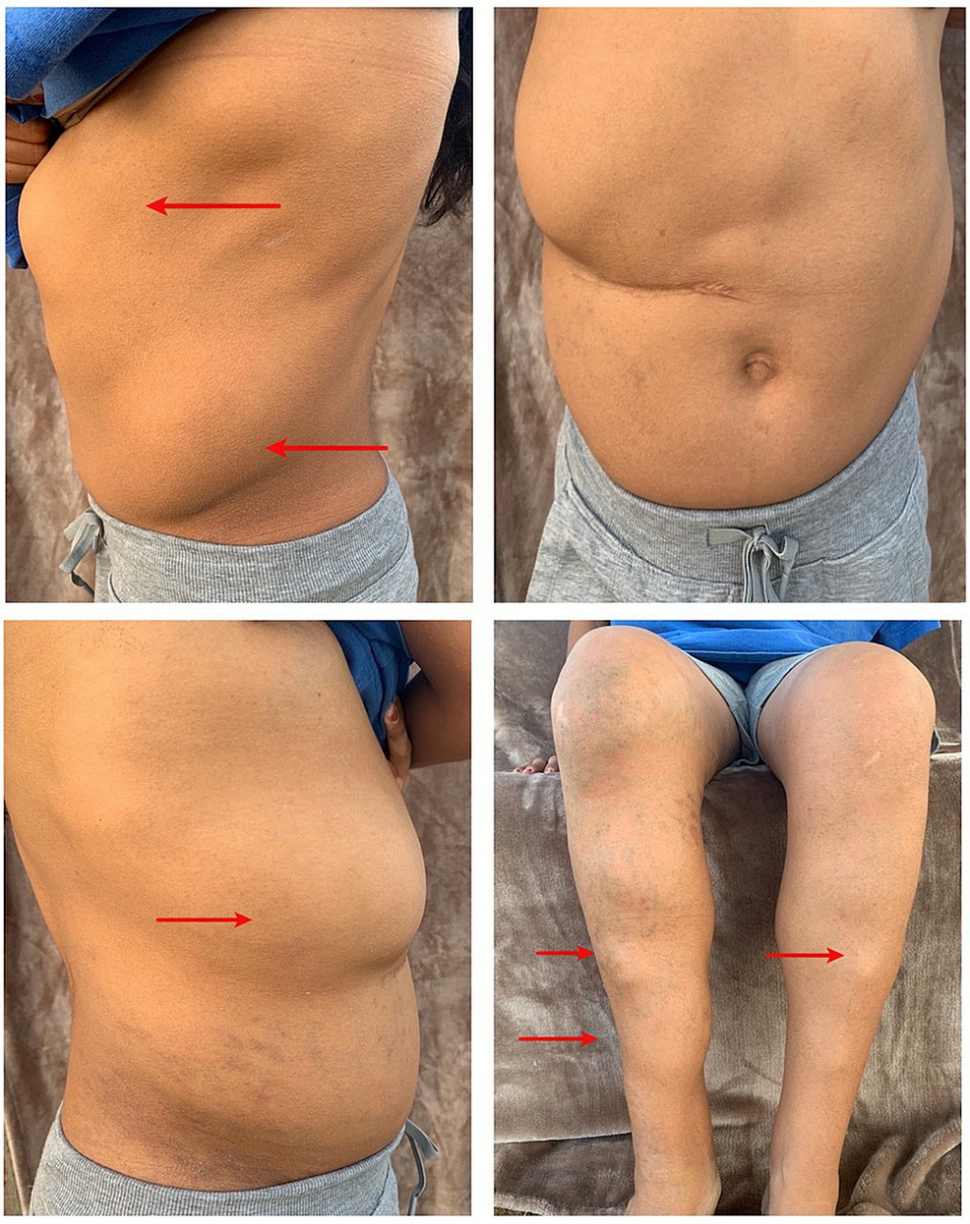
Multiple subcutaneous lipomas on the trunk, pelvis, and lower extremities.

A 25-year-old Hispanic female diagnosed with BRRS one year prior to presentation came in for burning epigastric pain over the past six months. The pain was intermittent, non-radiating, triggered by meals, relieved by antacids, and associated with postprandial nausea. She denied any weight changes, scleral icterus, hoarseness, cough, dysphagia, vomiting, diarrhea, constipation, hematochezia, or melena. The patient also denied frequent use of non-steroidal anti-inflammatory drugs (NSAIDs), cigarette smoking, or any history of *H. pylori* infection. The physical exam was relevant for a prominent head circumference above the 97th percentile, normal-looking facial features, mild epigastric tenderness, and multiple mobile soft tissue masses corresponding with lipomas of varying sizes. They were identified on the trunk, pelvis, and lower extremities (Figure 1).

The patient emigrated from Mexico when she was 19 years old. She was diagnosed with BRRS at the age of 24 years based on a history of neonatal macrocephaly, developmental delay, mild learning disabilities, and multiple subcutaneous lipomas which were identified at three years of age. The family history was unremarkable except for a diagnosis of colorectal cancer in her maternal grandmother at the age of 52 years.

None of the family members have any medical history of genetic disorders, developmental delay, or physical deformities. Based on the suggestive clinical findings and a molecular diagnosis that confirmed a heterozygous missense mutation in the PTEN gene, the patient was diagnosed with BRRS.

**Figure 2:**
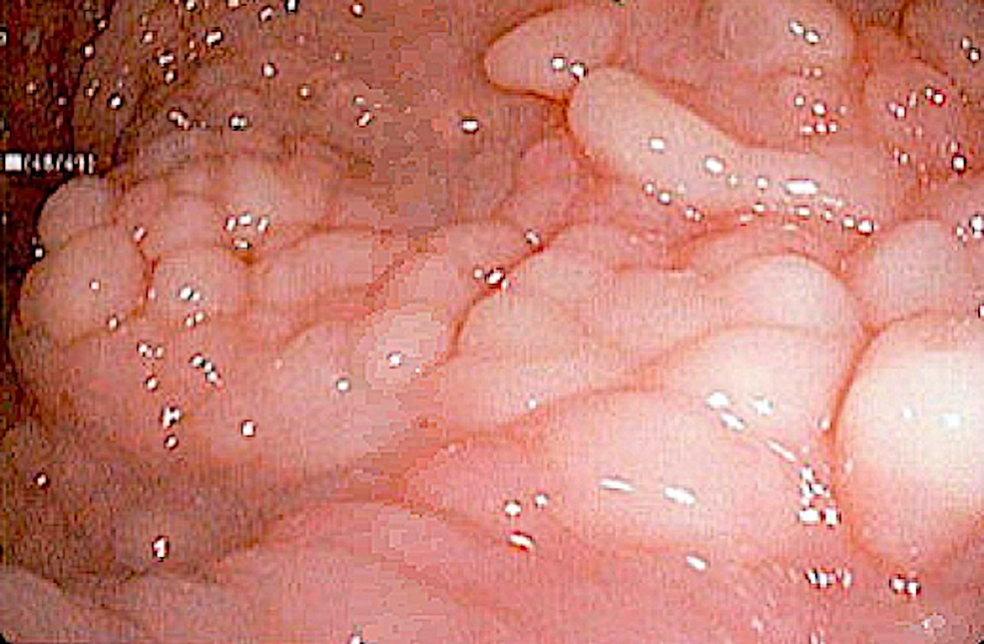
Endoscopic image of the distal esophagus showing sessile polyps.

**Figure 3:**
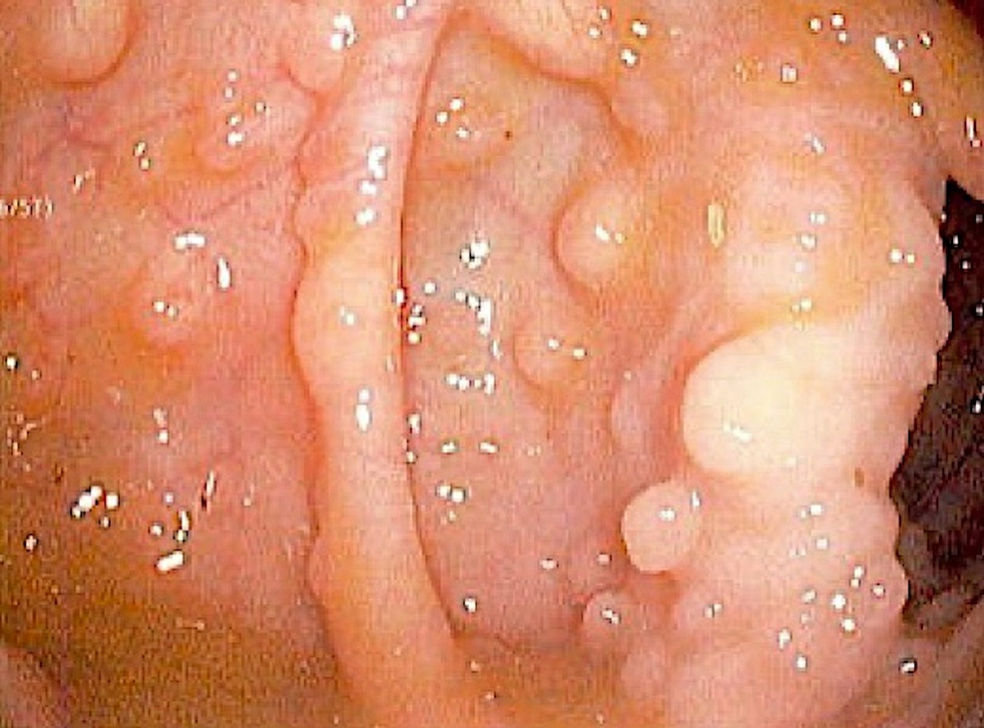
Endoscopic image of descending colon showing multiple sessile polyps.

Due to the increased risk of gastrointestinal cancer associated with this condition an endoscopic evaluation was performed. The esophagogastroduodenoscopy (EGD) revealed numerous 3-7 mm sessile polyps located in the distal esophagus and gastric cardia (Figure 2). An erythematous and erosive mucosa was observed in the gastric antrum. The duodenum appeared normal. The colonoscopy identified numerous 3-9 mm sessile polyps throughout the entire colon (Figure 3) and mild internal hemorrhoids. Histopathologic examination confirmed synchronous polyps of varying types, including inflammatory, lymphoid, and lipomas. Biopsy of the gastric mucosa identified chronic active gastritis and a positive immunohistochemical stain for *H. pylori*.

The patient received 14 days of concomitant eradication therapy for *H. pylori* infection. She also got education regarding BRRS, possible manifestations, and her risk of developing colorectal cancer and other malignancies. Four weeks after completion of treatment, infection eradication was confirmed by a urea breath test. She is currently following an age-appropriate cancer surveillance protocol with a multidisciplinary treatment team.

## Discussion

The PTEN gene, located on chromosome 10q23, is considered to be a tumor suppressor gene [3] that inhibits signaling pathways critical for cell proliferation, cell cycle progression (particularly in G1-S and G2-M transitions), and apoptosis [4]. The loss of function of this gene is associated with different cancers, including thyroid, colorectal, breast, endometrial, renal cancer, and melanoma [5-7]. Germline mutations in the PTEN gene have been described in a group of rare disorders collectively known as PHTS. The phenotypic spectrum of PHTS includes Cowden syndrome (CS), BRRS, Adult Lhermitte-Duclos disease, and autism spectrum disorders associated with macrocephaly [8].

Bannayan-Riley-Ruvalcaba syndrome has been shown to be a gene variant or allelic to CS, with approximately 60% of patients having PTEN mutations. At present, no other genes are known to cause BRRS [9]. The classic presentation occurs in the neonatal period or shortly thereafter with macrocephaly, subcutaneous lipomatosis, vascular malformations, and penile lentiginosis [6]. Other common clinical features include developmental delay, intellectual disability, and hamartomatous intestinal polyps [2]. There are no specific criteria for diagnosis but it is usually determined by the clinical presentation. A germline PTEN mutation confirms that the patient with this syndrome belongs to the PHTS group. Differential diagnoses include Lhermitte-Duclos syndrome, Juvenile polyposis syndrome, Peutz-Jeghers syndrome (PJS), Birt-Hogg-Dubé syndrome, Proteus syndrome, CS, Gorlin syndrome, and neurofibromatosis type 1 [9].

Recent data have shown that intestinal polyps are found in up to 95% of adults with PTEN mutations who have undergone colonoscopy [10]. Gastrointestinal polyps are distributed throughout the stomach, small and large intestine. The range of polyp types includes adenomas, ganglioneuromas, hamartomas, inflammatory polyps, leiomyomas, hyperplastic polyps, lipomas, and lymphoid polyps [10].

Although premalignant lesions were not thought to be a feature of this syndrome; it is now believed that BRRS patients with a germline PTEN mutation share the same risk of cancer development as CS patients [2]. There are no consensus guidelines for cancer surveillance in patients with this syndrome. It is suggested to follow the CS/PHTS cancer surveillance recommendations if a PTEN pathogenic variant is present (Tables 1-2) [11].

**Table 1:** PTEN hamartoma tumor syndromes cancer surveillance recommendations. PTEN, phosphatase and tensin homolog

Starting age	Recommendation	Frequency
7	Thyroid ultrasound	Annual
18	Dermatologic evaluation	Annual
35 (unless symptomatic)	Colonoscopy	Every five years (more frequently if symptomatic or polyps are noted).
			40	Renal ultrasound	Every one to two years

**Table 2:** Additional recommendations specifically for women.

Starting age	Recommendation	Frequency
18	Breast self-examination	Monthly
30-35	Mammography and breast MRI	Annual
35	Random endometrial biopsies and transvaginal ultrasound	Annual

The lifetime risk of developing colorectal cancer among patients with PTEN mutations is estimated at 9%. Current guidelines suggest the endoscopic resection of gastrointestinal polyps given the varied histology, including frequent adenomatous polyps and the risk for dysplastic change and colorectal cancer. Endoscopic techniques to improve visual differentiation of polyp histology, such as narrow-band imaging, should be considered to allow for preferential removal of large polyps (≥1 cm) or those that appear to be adenomatous [12].

Future treatment options may involve targeting the genetic pathways affected by the loss of the PTEN gene function [2]. In a previous clinical trial with sirolimus, some participants had a reduction in the number of colon polyps with its use for a short period. Although these results are intriguing, it is clear that further data are needed regarding the potential benefits and risks of this therapy before widespread usage. Currently, an open-label pilot trial is being conducted to determine whether sirolimus reduces colon polyp burden in patients with a confirmed mutation in the PTEN gene [13-14].

## Conclusions

Bannayan-Riley-Ruvalcaba syndrome is an autosomal dominant disorder caused by germline mutations in the PTEN gene. Early recognition of this syndrome is critical given the increased risk of different cancers, including thyroid, colorectal, breast, endometrial, renal cancer, and melanoma.

Although our patient was already diagnosed with this syndrome, the case illustrates the importance of a detailed clinical examination to associate PTEN gene mutations with multiple subcutaneous lipomas, macrocephaly, and intellectual disability. It is crucial to individualize colorectal cancer surveillance recommendations based on personal and familial history. A colonoscopy should begin at the age of 35 years or before if the patient is symptomatic.

We recommended the patient perform monthly breast self-examinations and undergo screening for thyroid cancer and melanoma. Patients should begin screening for breast cancer, endometrial cancer, and renal cell carcinoma at a subsequent time. This disorder affects multiple organ systems, and close follow-up with a multidisciplinary treatment team has been arranged, including dedicated genetic counseling.
